# Surgical refinements and sensory and functional outcomes of using thinned sensate anterolateral thigh perforator flaps for foot and ankle reconstruction: A retrospective study

**DOI:** 10.1097/MD.0000000000038763

**Published:** 2024-09-13

**Authors:** ZhaoHui Pan, YuXiang Zhao, XingHua Ye, JianBo Wang, XingBo Li

**Affiliations:** a Institute of Orthopedic Trauma Surgery of the Chinese People’s Liberation Army, 80th Group Military Hospital, Weifang, China.

**Keywords:** foot reconstruction, lateral femoral cutaneous nerve, sensate anterolateral thigh perforator flap, sensory test, thinned flap

## Abstract

To improve the use of sensate anterolateral thigh (ALT) flaps for foot and ankle reconstruction, we employed a thinned nerve-selective harvesting technique. The data of 31 patients in whom sensate ALT perforator flaps were transferred for reconstruction of soft-tissue defects in the foot and ankle were reviewed. Flaps were elevated with 2 refinements. The first is the initial selection of the “true” sensory branch in the medial incision on the suprafascial plane. The second is flap thinning by keeping a cuff of thin deep fat surrounding the point where the perforator or nerve branch inserts into the superficial fat layer. The recipient site assessment consisted of complications, monofilament touch perception, sharp–blunt discrimination, axial circumference, and American Orthopedic Foot and Ankle Society score. After a mean follow-up of 31.7 months, all flaps survived uneventfully, except for marginal necrosis in 1 patient, infection in 1 patient, ulceration in 2 patients, and secondary thinning in 3 patients. The sensation of each flap was restored. A total of 87% and 90% of the patients exhibited 5 or more positive response points in the Semmes–Weinstein monofilament touch and sharp–blunt discrimination testings, respectively. The mean axial circumference of the reconstructed foot was 27.4 cm (the unaffected side was 25.8 cm). All patients achieved mobility in ordinary shoes with a mean functional score of 74.6. The thinned nerve-selective sensate ALT perforator flap can be a favorable option for foot and ankle reconstruction. This method also offers the possibility of preserving the nerve branch at the donor thigh.

## 1. Introduction

The sensate anterolateral thigh (ALT) perforator flap has been increasingly used for the reconstruction of large soft-tissue defects in the foot and ankle,^[1–6]^ although the benefit of performing nerve coaptation remains controversial. This success is mainly based on its reliability for harvesting nourish vessels, versatility in flap design, ease of dissecting sensory nerves, feasibility for allowing a two-team approach and acceptable donor-site morbidity. However, some limitations may hinder its use in foot and ankle reconstruction. First, the excess subcutaneous fat often has to be removed from the skin flap to be appropriate for resurfacing shallow wounds.^[7,8]^ In this scenario, small branches of the lateral femoral cutaneous nerve (LFCN) supplying the flap, which are hard to distinguish from fascia tissue after flap elevation, may be disrupted because of their intra-adiposal distribution in the flap. Therefore, flap sensation can be damaged. This is somewhat similar to the blood vessels in the adipose layer, which can sometimes also be jeopardized by a thinning procedure. Second, permanent sensory deficits in the distal donor thigh sometimes develop due to direct neurotomy of the main trunk of the LFCN in the conventional sensate flap harvesting technique.^[9,10]^ To improve the use of sensate ALT perforator flaps in foot and ankle reconstruction, we modified the flap harvesting technique. The clinical results, sensory recovery of the flaps, foot axial circumflex and function were presented.

## 2. Patients and methods

From January 2015 to December 2020, patients who underwent ALT perforator flap transfers for foot and ankle wounds in our hospital were enrolled in this retrospective study. The inclusion criteria included patients older than 18 years and primary thinned sensate ALT perforator flap transfer for foot and ankle wounds performed by the first author (Z.H. Pan). Exclusion criteria included loss to contact, declined to participate due to personal matters, not undergoing a primary thinning procedure, bilateral reconstruction and missing follow-up data. The study was reviewed and approved by the Ethics Committee of the 80th Group Military Hospital. Written consent was obtained from the patients for the purpose of publication of case details, images, and videos.

Data were collected regarding age at the time of surgery, sex, body mass index (BMI), etiology, involved subunit,^[11]^ defect type according to the Hidalgo and Shaw definition,^[12]^ flap design, skin paddle length and width, and flap thickness before and after thinning.

**Figure 1.**
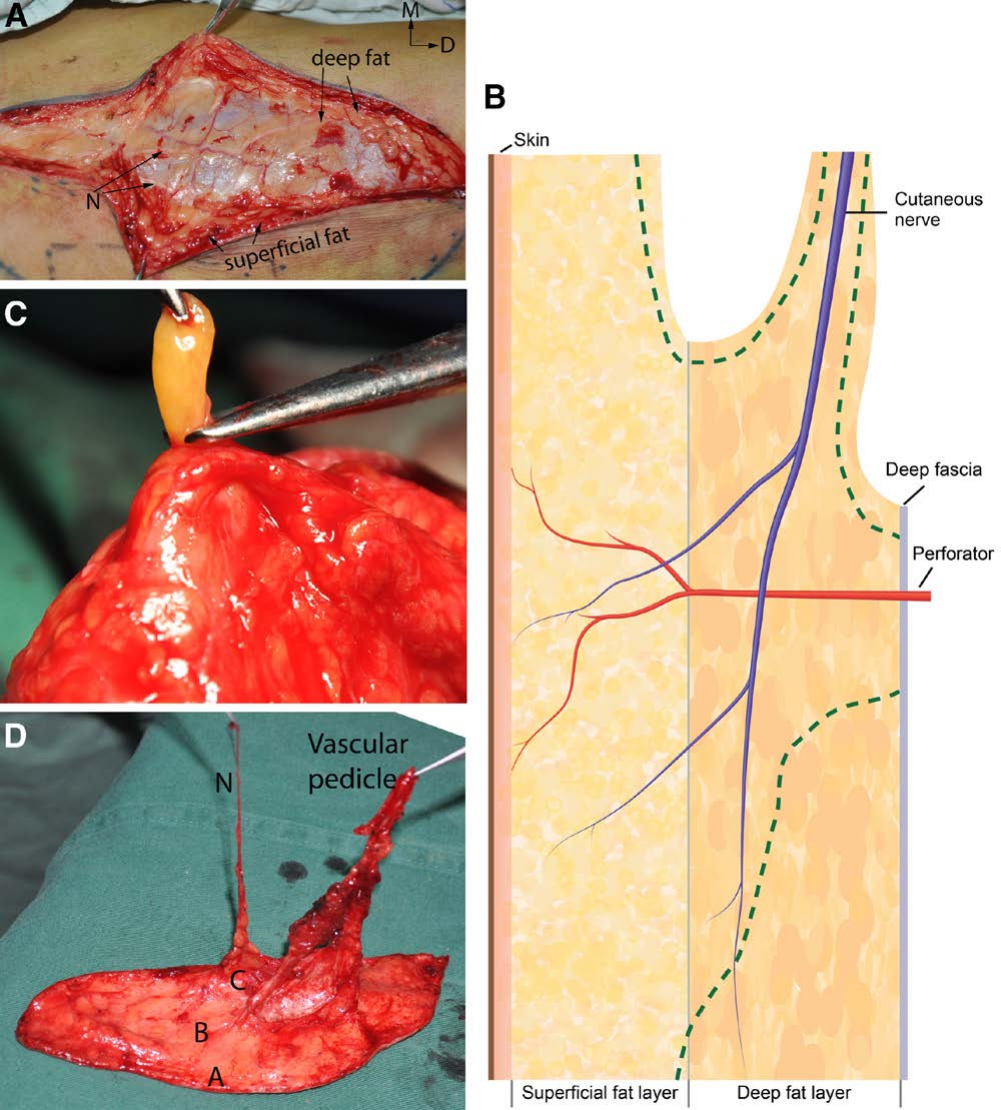
(a) Two subbranches of the lateral femoral cutaneous nerve are shown in the deep fat layer. The lateral subbranch, running toward the anterior aspect of the flap, is selected as the “true” sensory nerve, while the medial subbranch is spared at the donor thigh. (b) A schematic drawing showing how to protect the vascular perforator and sensory branch during flap elevation (vertical view). The green dashed line indicates the dissection plane. (c) Removal of the fat lobule in the deep layer of fat repetitiously after flap elevation. (d) Appearance of a sensate ALT flap including a domain sensory nerve after thinning and transection of the pedicle. (A) The skin edge of the flap; (B) superficial fat layer; (C) a cuff of thin deep fat; (N) a branch of the lateral femoral cutaneous nerve.

A medial incision was first made approximately 3 cm medial to the line from the anterior superior iliac spine to the superolateral corner of the patella to identify the superficial fascia layer, after which the dissection proceeded laterally. The branch of the LFCN and its subbranch carrying sensation for the flap were found and selected by meticulous dissection between the deep and superficial fat layers (Fig. 1a and Video 1, Supplemental Digital Content, http://links.lww.com/MD/N539 and Video 2, Supplemental Digital Content, http://links.lww.com/MD/N540). Then, the point where the main perforator inserted into the superficial fat was identified. Once this information was known, the flap was elevated toward the main perforator at the superficial fascia level while maintaining an area of 1.5 cm in the radius of deep fat and deep fascia around the perforator (Fig. 1b). Then, the proximal deep fascia was opened, and the vascular pedicle was released. Further thinning was performed prior to ligation of the vascular pedicle (Fig. 1c), keeping a cuff of thin deep fat surrounding the perforator or nerve branch (Fig. 1d). If needed, the flap could include the deep fascia, vastus lateralis muscle or vascular conduit of the descending branch of the lateral circumflex femoral vessel in varying proportions.

The recipient site assessment consisted of postoperative complications (flap necrosis, infection, ulceration and secondary thinning), touch perception (10 g Semmes–Weinstein monofilament), and sharp–blunt discrimination (sharp and blunt pin) sensory testing, axial circumferences of the reconstructed and contralateral feet,^[13]^ and American Orthopedic Foot and Ankle Society (AOFAS) scale score.^[14]^ Sensory tests were performed at 8 predetermined points sampled from 4 zones over the whole flap. The results were expressed as the ratio of the number of positive response points to the 8 tested points on each flap.

## 3. Results

A total of 31 patients who underwent 31 thinned nerve-selective sensate ALT perforator flap transfers were included. The mean age at the time of surgery was 47.1 ± 11.5 years. The vacuum sealing drainage technique was applied to traumatic defects for 5 to 7 days before reconstructive surgery. The dorsal foot was the most common affected subunit. Twenty-six flaps included 1 sensory branch (a domain branch in 19, a subbranch divided from a domain branch in 5, and a small branch in 2), and 5 flaps included 2 sensory branches (a domain branch and a small branch). End-to-end neurorrhaphy was performed on the sensory nerve adjacent to the wound.

A follow-up examination was performed at 31.7 ± 22.4 months. One patient developed marginal necrosis at the proximal tip of the flap, which healed with dressing, and another patient developed an infection, which was treated with dressing, debridement and systemic antibiotic therapy. Ulceration developed in the plantar area of the flap in 2 patients within 6 months postoperatively, and resolved spontaneously with offloading. No recurrence was detected throughout the follow-up period. Secondary thinning was performed in 3 patients, which was postponed to more than 1 year after sensory recovery was evaluated. The sensation of each flap was restored. Furthermore, a total of 87% (27/31) and 90% (28/31) of the patients exhibited 5 or more positive response points in the Semmes–Weinstein monofilament touch and sharp–blunt discrimination testings, respectively. Regarding the axial circumference, an independent sample *t* test revealed that the reconstructed and contralateral feet differed significantly (27.4 ± 3.0 vs 25.8 ± 2.6 cm, t = 2.299, *P* = .025 < .05). All patients achieved mobility in ordinary shoes, and the mean AOFAS score was 74.6 ± 10.1 (Table 1).

**Table 1:** Demographic, operative data, and outcomes.

Case	Age (yr)/Sex	BMI	Etiology	Location	Defect type	Flap design	Size of skin paddle (cm)	Thickness I/F (mm)	complications	F/U (ms)	Sensory test		AC (C) (cm)	AOFAS score
											SW	SB		
1	65/M	22.0	Crush injury	L s.unit5	III	Fc	21 × 9	10/5	None	12	6/8	7/8	25.2 (23.4)	82
2	47/M	25.9	Crush injury	L s.unit 5	III	Fc	18 × 7	12/4	None	60	6/8	5/8	27.7 (25.1)	88
3	53/M	29.1	TA	R s.unit 5 + 6	III	Fc	22 × 9	16/5	Marginal necrosis	12	6/8	7/8	27.9 (26.2)	70
4	50/F	21.7	TA	R s.unit 6 + 7	III	chimeric	15 × 9	12/5	None	18	6/8	7/8	26 (25)	60
5	58/M	23.0	Blast injury	R s.unit 2 + 3 + 5	III	flow through	20 × 9	15/5	Ulceration	12	6/8	5/8	24.3 (30.5)	59
6	40/M	25.2	Crush injury	R s.unit 4 + 6 + distal leg	III	flow through	20 × 9	13/5	None	24	8/8	8/8	30.8 (28.3)	75
7	60/F	25.0	Crush injury	L s.unit 5 + 6	II	Fc	20 × 10	15/5	None	60	7/8	7/8	30.7 (27.2)	85
8	44/M	21.5	Crush injury	L s.unit 6 + 7 + distal leg	III	flow through	22 × 9	10/5	None	24	8/8	8/8	31.8 (30.5)	94
9	45/M	24.2	Crush injury	L s.unit 1 + 5	III	chimeric	17 × 8	12/5	None	72	5/8	5/8	26.9 (24.5)	91
10	43/M	22.8	Crush injury	R s.unit 1 + 5	III	chimeric	15 × 9	12/4	None	24	7/8	7/8	25.5 (24.7)	65
11	43/M	26.8	Crush injury	R s.unit 3 + 4	III	chimeric	20 × 10	14/5	None	72	4/8	3/8	24 (23)	79
12	58/M	27.1	Crush injury	R s.unit 1 + 5 + 6	II	Fc	18 × 8	12/5	None	24	8/8	7/8	26.9 (24.2)	90
13	49/M	23.7	Crush injury	L s.unit 3 + 4 + 6	II	chimeric	15 × 8	10/4	None	24	7/8	7/8	34.2 (30.6)	79
14	48/F	40.2	Crush injury	L s.unit 5 + 6	II	Fc	20 × 10	18/6	Secondary thinning	84	5/8	5/8	29.7 (26.3)	72
15	38/F	22.2	TA	R s.unit 4 + 5 + 6	II	chimeric	24 × 9	16/6	None	72	8/8	5/8	24.1 (22.3)	73
16	25/F	20.6	TA	L s.unit 1 + 5 + 6 + distal leg	III	Fc	26 × 8	15/6	None	12	4/8	5/8	25.4 (23.5)	70
17	41/F	31.2	TA	R s.unit 5	III	chimeric	25 × 8	10/4	Secondary thinning	12	5/8	5/8	26.8 (24.3)	63
18	27/M	27.8	TA	L s.unit 6 + 7	III	flow through	20 × 10	15/5	None	48	3/8	4/8	31.6 (28.5)	74
19	65/F	18.7	TA	L s.unit 5 + 6	II	Fc	14 × 9	13/4	None	48	4/8	7/8	27.3 (25.7)	65
20	52/M	23.7	Blast injury	R s.unit 2 + 5	III	chimeric	22 × 10	13/5	None	18	8/8	8/8	28 (25.2)	77
21	41/M	23.0	Crush injury	R s.unit 2 + 5	III	flow through	20 × 10	11/5	None	24	6/8	5/8	24.9 (27)	75
22	37/M	23.2	Crush injury	R s.unit 3 + 5	III	Fc	15 × 8	13/5	None	24	7/8	8/8	25.8 (25.1)	74
23	53/M	22.5	Tumor excision	L s.unit 4	II	Fc	20 × 8	10/5	None	12	7/8	8/8	30.5 (30)	91
24	58/M	20.3	TA	R s.unit 5	II	Fc	20 × 8	7/4	None	12	6/8	7/8	24.3 (24)	88
25	18/M	23.3	Crush injury	L s.unit 2 + 5	III	Fc	20 × 10	12/4	None	24	8/8	7/8	25.9 (24.1)	70
26	32/M	23.6	Crush injury	L s.unit 1 + 5	III	Fc	20 × 10	12/6	None	24	6/8	6/8	26.9 (25.2)	68
27	44/M	30.1	Crush injury	R s.unit 1 + 2 + 5	III	chimeric	17 × 8	13/4	Secondary thinning	24	7/8	8/8	24.7 (24)	74
28	52/F	23.0	TA	R s.unit 3	III	Fc	16 × 10	16/4	None	60	5/8	4/8	24.8 (22.3)	73
29	55/M	23.7	Crush injury	L s.unit 1 + 5	III	flow through	24 × 8	10/4	None	18	6/8	6/8	27.5 (24.1)	71
30	61/M	22.6	TA	L s.unit 3 + 5 + 6	III	flow through	25 × 9	6/4	None	12	8/8	6/8	24.5 (23.3)	60
31	57/M	20.8	Crush injury	R s.unit 6	III	chimeric	20 × 9	12/5	Infection	18	6/8	5/8	34.5 (30.8)	59

AC = axial circumference, AOFAS = American Orthopedic Foot and Ankle Society, C = contralateral foot, F = female, F = final thickness, F/U = follow-up, Fc = fasciocutaneous flap, I = initial thickness, L = left, M = male, R = right, s.unit = subunit, SB = sharp–blunt discrimination, SW = Semmes–Weinstein monofilament, TA = traffic accident.

## 4. Case report

**Figure 2.**
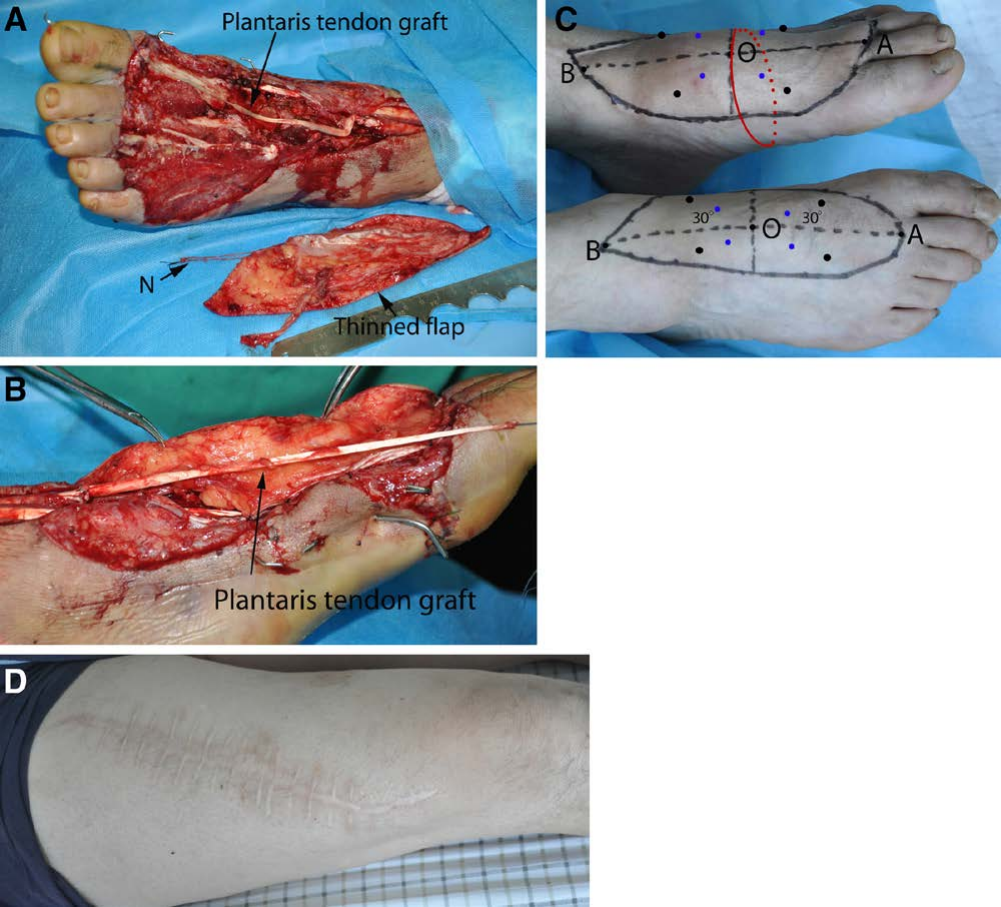
A 47-year-old male patient with a crush injury to the left foot dorsum. (a) Wound with soft-tissue and extensor hallucis longus defects after the second debridement and a harvested ALT flap (15 mL of subcutaneous fat was removed). (N) 2 branches of the lateral femoral cutaneous nerve. (b) Insetting the grafted tendon between the fascia lata and the superficial fat layer of the flap. (c) Good contour of the foot at 60 months postoperatively. The photograph also demonstrates the orientation of the 8 predetermined points for sensory testing and circumference measurement along the horizontal axis (red line) on both feet. The horizontal axis of the flap is drawn across midpoint O of the longitudinal axis connecting points. (A and B) Each flap is then divided into 4 zones. Eight testing points are then determined at the junctions of the medial-middle and lateral-middle thirds of the 2 30° angular sector lines connecting point O with the flap margin. (d) Good contour of the donor thigh at 60 months postoperatively.

A 47-year-old man sustained a crush injury to his left foot. Five days after initial debridement, internal fixation and negative pressure coverage, reconstruction was performed with a thinned sensate ALT perforator flap that included a strip of deep fascia and 2 nerve branches (Fig. 2a). After the exposed bone and joints were covered with deep fascia, the defect of the extensor hallucis longus was bridged with a graft of the plantaris tendon (Fig. 2b). Then, the skin paddle was positioned on the defect. The sensory branches were coapted to the saphenous nerve. The follow-up at 60 months showed good foot (Fig. 2c) and donor thigh contours (Fig. 2d). The ability to discern Semmes–Weinstein monofilament touch and sharp–blunt discrimination was observed for 6 of 8 points and 5 of 8 points, respectively.

## 5. Discussion

**Figure 3.**
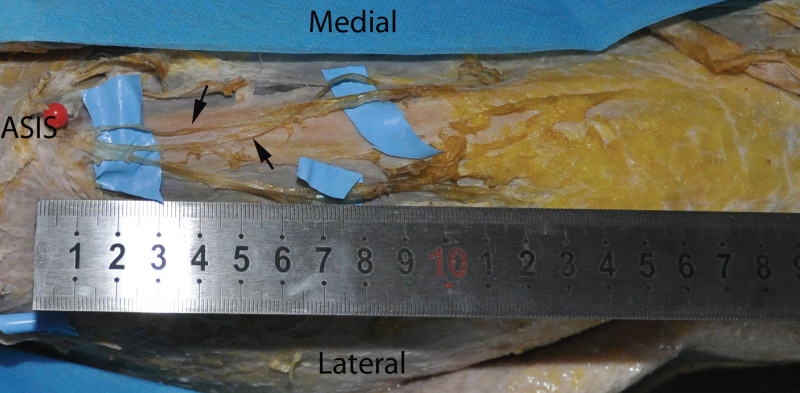
A cadaver dissection on the right side. The proximal part of the anterior thigh is viewed to show the branches of the LFCN. Two subbranches (arrows) can be spared at the donor site. ASIS: anterosuperior iliac spine.

The issue of donor nerve selection and its location in sensate ALT flaps requires further investigation. The anterior branch of the LFCN has been described as the domain cutaneous nerve supplying sensation to the ALT flap. It usually lies along a line connecting the anterior superior iliac spine and superolateral patella.^[15–17]^ Along the vertical axis of the thigh, the anterior branch lies in the deep fat layer in the proximal thigh and then gradually approaches the superficial fat layer as it runs distally.^[16]^ Its diameter and course are variable in the proximal thigh.^[18]^ In this series, the anterior branch, which is usually inserted in the middle of the skin paddle, was used as the dominant nerve for the whole flap. The proximal portion of the flap might be innervated by the posterior branch of the LFCN. All these nerves might share territory via anastomoses between them. In this study, we present 2 refinements to improve the use of sensate ALT perforator flaps for foot and ankle reconstructions. First, the anterior branch of the LFCN was initially dissected via a medial incision. In comparison with the usual procedure of dissecting the sensory nerve after all sides of the ALT flap are elevated, initial nerve dissection enables a clear view of the nerve course and its sprouting fascicles. Thus, this modification allows the surgeon to select the “true” sensory branch to the skin paddle. This procedure is similar to the microdissection thinning technique, which enables the vascular distribution in the adipose tissue to be observed directly. Additionally, both the superficial and deep layers of the adipose tissues surrounding the sensory branch can be removed simultaneously. Sensory deficit in the donor thigh was the most common symptom after raising the ALT flap. Securely preserving the nerve during flap harvesting can prevent sensory deficits at the donor site.^[10]^ Additionally, coaptation of the distal part of the LFCN end to side to any of the nearby sensory nerves could preserve some sensation at the donor thigh.^[6]^ Although our clinical experience and the limited number of cadaver dissections (Fig. 3) prevented us from determining the actual frequency of LFCN innervation pattern variation, we believe that in a certain portion of patients, some branches of the LFCN could be spared at the donor thigh, which could substantially reduce the risk of sensory deficits at the donor thigh. The second is preserving a thin deep fat surrounding the perforator or nerve branch. Thus, flap thinning will do less harm to vessels and nerves. Occasionally, when nerve visualization cannot be achieved in the proximal region because of its variable course, the distal border of the flap, where the fat layer is relatively thin, can be dissected to search for nerves. From there, gently pulling the distal part of the branch could be applied to expedite tracking of the nerve to its proximal part.

Although previous studies have reported early sensory recovery following the use of the sensate ALT flap in foot and ankle reconstruction, the sample of patients was small, and the test site was rarely mentioned (Table 2). Our report not only included the largest number of patients who had undergone surgery with a sensate ALT flap but was also the first report evaluating sensory recovery by 2 sensory modalities at 8 representative points distributed in 4 zones over a whole flap. Monofilament touch perception reflects the function of large myelinated A-β fibers, while sharp–blunt discrimination reflects the function of small A-δ fibers.^[19]^ When 5 or more of the 8 points were recognized, the positive response rates of 87% and 90% in the 2 modalities indicated that primary thinning did not have a negative effect on nearly uniform sensory and synchronous reinnervation in the flap. Attempts at comparison with previous reports were difficult due to the lack of a commonly accepted assessment method. It is possible that the best focal testing result previously reported in a certain area of flap might have been recorded as the final outcome. Notably, we found that in most flaps in this series, sensation did not recover to the level of normal skin. This discrepancy might result from inconsistent neurosomal boundaries and needs further research.

**Table 2:** Published sensory recovery of sensate ALT flaps for foot defects.

Author	Number	Area reconstructed	Flap size (cm^2^)	F/U (mo)	, Sensory modalities and results
Yildirim et al^[1]^	4	Amputation stump	N/S	N/S	2PD, 22–28 mm (100%)
Hong et al^[2]^	6	Plantar aspect	N/S	12	SWM touch, 6 (100%)
Olivan et al^[3]^	7	Plantar aspect	12–50	12	1 PS, 8.1–11.2 g/mm^2^; 1 PM, 9.4–13.4 g/mm^2^
Pappalardo et al^[4]^	12	Weighting bearing sole	100–300	10–48	SWM touch, 12 (100%); 2PD, 12–15 mm (100%)
Xie et al^[5]^	12	Dorsum, ankle, distal leg	20–102	10–24	SWM touch, 12 (100%); 2PD, 13–16 mm (100%)
Ellabban et al^[6]^	6	Heel	N/S	12	SWM touch, 6 (100%)
Present study	31	Foot, ankle	120–220	12–84	SWM touch, 27 (87%); SBD, 28 (90%)*

1 PM = moving one-point test, 1 PS = static one-point test, 2PD = two-point discrimination, F/U = follow-up, N/S = not stated, SBD = sharp–blunt discrimination, SWM = Semmes–Weinstein monofilament.

* 5 or more positive response points over a whole flap were recognized.

Ideally, the thickness of the flap should be individually thinned according to the demand for a given subunit to achieve a bespoke three-dimensional inset. Regarding the subunit of the dorsal foot and ankle resurfacing, the optimal thickness of an ALT flap should be approximately 3 mm. However, there is no consensus as to the most suitable thickness when an ALT flap is arranged to the weight-bearing subunit of the foot. Hong and Kim reported that an ALT flap composed of thin superficial fat with a thickness of 4 to 6 mm was appropriate for plantar reconstruction to reduce the need for a secondary thinning procedure and improve stability.^[2]^ Hollenbeck et al noted that thin flaps might be subject to breakdown in weight-bearing subunits. They also suggested that aesthetic principles should be integrated to maximize both function and form.^[20]^ Regarding the weight-bearing subunit, we performed ALT flap thinning by removing the deep fat tissue while maintaining the superficial fat tissue with a thickness of approximately 5 mm to prevent bulky and sideways movement. Due to the inclusion of a cuff of thin deep fat surrounding the perforator or nerve branch, a small amount of excess fat tissue might remain at the central portion of the flap. Since the BMI of most patients in this series was within the normal weight or overweight range, it was not difficult to adapt these thinned flaps to foot and ankle defects. However, the mean axial circumference was slightly greater than that of the unaffected foot. We agree with Kwon JG, et al that in patients with a high BMI, ALT flaps elevated from the superficial fat level may still not be thin enough and may require secondary thinning.^[21]^ In this series, 3 patients with a BMI > 30 kg/m^2^ underwent secondary thinning. Interestingly, 2 patients with inadequate sensory recovery developed ulceration in the weight-bearing area within 6 months postoperatively. This was in accordance with a report by Struckmann et al, who reported that 3 patients treated with different flap types who achieved different amounts of sensory recovery developed ulcerations in the reconstructed heel.^[22]^ In our opinion, in addition to sensation considerations, flap thickness and design (absorbing impact during walking),^[4,23]^ flap stability (adhering to deep structures for resistance to shear force),^[3]^ as well as patient education and compliance^[2]^ are some of the reasons for the occurrence of ulceration in the weight-bearing area.

The limitations of our study include the small sample size, the nonrandomized retrospective nature of the study, and the insufficient power to provide definitive conclusions. Furthermore, other factors that may affect the quality of sensory recovery, such as the choice of recipient nerve, were not evaluated. Additionally, owing to the heterogeneity of coexisting injury and flap design, donor site morbidity and the duration of the surgical procedure were not evaluated. Finally, the operation time was extended by approximately 30 minutes for nerve dissection and coaptation.

## 6. Conclusion

A thinned nerve-selective sensate ALT perforator flap can be a favorable option for foot and ankle reconstruction. This method also offers the possibility of preserving the nerve branch at the donor thigh.

## Acknowledgments

The authors thank Xiaoming Ren for the support of the statistical analysis.

## Author contributions

**Conceptualization:** ZhaoHui Pan.

**Data curation:** YuXiang Zhao, XingHua Ye, JianBo Wang, XingBo Li.

**Methodology:** YuXiang Zhao, XingHua Ye, JianBo Wang, XingBo Li.

**Writing – original draft:** ZhaoHui Pan.

**Writing – review & editing:** ZhaoHui Pan, YuXiang Zhao, XingHua Ye, JianBo Wang, XingBo Li.

## Supplementary Material


